# lncRNA ANRIL aggravates the chemoresistance of pancreatic cancer cells to gemcitabine by targeting inhibition of miR-181a and targeting HMGB1-induced autophagy

**DOI:** 10.18632/aging.203251

**Published:** 2021-08-10

**Authors:** Lei Wang, Rongrong Bi, Lei Li, Kun Zhou, Hang Yin

**Affiliations:** 1 Department of Gastroenterology, Shanghai Ninth Peoples’ Hospital, Shanghai Jiaotong University, Shanghai 200011, China; 2 Department of Pulmonary, Longhua Hospital, Shanghai University of Traditional Chinese Medicine, Shanghai 200032, China

**Keywords:** lncRNA ANRIL, miR-181a, HMGB1, pancreatic cancer

## Abstract

Recent studies focus on long noncoding RNAs (lncRNA) as crucial regulators of cancer biology that contribute to essential cancer cell functions such as cell proliferation, apoptosis, and metastasis. In pancreatic cancer, several lncRNAs have been mentioned as important actors in tumorigenesis. However, the function of lncRNA ANRIL (named as ANRIL as follows) in pancreatic cancer has not been elucidated. In the present study, we show that ANRIL was up-regulated while miR-181a was down-regulated in pancreatic cancer tissues and HMGB1 was highly expressed. Knockdown of ANRIL in pancreatic cancer repressed cellular proliferation, invasion, migration, and reduced chemotherapy resistance to gemcitabine. ANRIL was negatively correlated with miR-181a, while overexpression of miR-181a could reverse the effect. For further mechanism research, we found that miR-181a aimed to HMGB1 which activated cell autophagy. Taken together, our results implicate that the ANRIL, by targeting miR-181a, activates the HMGB1-induced cell autophagy, which is thought to be critical for oncogenesis.

## INTRODUCTION

Pancreatic cancer (PC) as a kind of common and frequent malignancy across the world, which may the major causes of cancer mortality in developed countries [1, 2]. Pancreatic cancer is still a destructive malignant disease although the progress development of surgical techniques and adjuvant drugs. Its median survival time is about 3–6 months and the five-year survival rate is less than 5% [3]. The morbidity of pancreatic cancer in China is lower compared with western countries but it has increased high in recent years. It has been reported that there are about 34509 men and 23226 women that die in pancreatic cancer, and the number of deaths exceeded that of the United States [3]. Therefore, this malignant tumor tends to be in late stage after definite diagnosis due to progress rapidly [4]. About 15–20% patients were diagnosed as resectable PC because of these factors and characteristic of specific anatomy, but most of them were diagnosed as locally advanced or metastatic disease with poor prognosis [5]. Therefore, it is better to learn the mechanism of PC and identify the innovative biological targets.

Long non-coding RNAs (lncRNAs) are transcripts more than 200 nucleotides in the length which have no obvious protein-coding functions [6]. ANRIL, namely CDKN2B-AS), is a long-chain non-coding RNA transcribed from the CDKN2A / B gene cluster on the Chr9p21 chromosome and plays an important role in tumor formation. Researches have indicated that the expression of ANRIL is significantly increased in nasopharyngeal carcinoma [7], and it also promotes cancer development, for example, lung cancer [8], and gastric cancer [9].

miR-181a has already been demonstrated to be an inhibitor of miRNA in many malignancy, such as breast cancer [10], oropharyngeal cancer [11], etc. However, the activity and role of miR-181a in tumor needs further study. HMGB1, as one of the targets of miR-181a, exhibits an essential effect on the inflammatory response [12], and also has stimulative effects in carcinogenesis [13].

In this research, the effect of ANRIL and miR-181a on pancreatic cancer had been revealed. It found that ANRIL was up-regulated, but miR-181a was inhibited in the tissues of pancreatic cancer. The inhibition of ANRIL could restrain the cell proliferation, invasion, migration of pancreatic cancer, and reduce chemotherapy resistance to gemcitabine through sponging miR-181a to target HMGB1-induced cell autophagy. This research illustrated the mechanism of ANRIL and miR-181a in the occurrence and development of pancreatic carcinoma, which might provide a novel strategy for pancreatic carcinoma therapy.

## MATERIALS AND METHODS

### Cell culture and reagents

Human pancreatic cancer cell lines PANC-1 (ATCC^®^ CRL-1469), BxPC-3 (ATCC^®^ CRL-1687) and HPDE were purchased from the American Type Culture Collection (ATCC) and cultured in Gibico’s RPMI-1640 Medium (Gibco, Invitrogen, Thermo Fisher Scientific, USA), with 10% fetal bovine serum (Gibco, Invitrogen, Thermo Fisher Scientific, USA), 1% penicillin (Gibco, Invitrogen, Thermo Fisher Scientific, USA) and streptomycin (Thermo Fisher Scientific, Gibco, Invitrogen, USA).

### Cell proliferation assay

Cells were seeded into 96-well plates for 4 hours before detection. Normal cell culture medium was replaced by fresh medium which contained 0.5% MTT (Abcam, USA). Dimethyl sulfoxide (DMSO) dissolved formazan (Abcam, USA) in cells and determine its light absorption value at 570 nm using an enzyme-linked immunosorbent detector (Invitrogen, Thermo Fisher Scientific, USA). Relative absorbance value indirectly reflects the number of living cells.

### Western blot

Proteins were obtained from cell lysates supernatants that using RIPA buffer (Invitrogen, Thermo Fisher Scientific, USA) supplemented with Phenylmethanesulfonyl fluoride (Sigma, USA). Denatured protein (30 μg) was resuspended and separated by 12% sodium dodecyl sulfate polyacrylamide gel. Then blotted onto nitrocellulose membrane (Bio-Rad, USA) at 300 mA for 1 h. Sequentially incubate primary and secondary antibodies on nitrocellulose membrane. Finally, protein bands exposed by bioimager (Bio-Rad, USA) with western blot detection kit.

### Quantitative real-time PCR (qRT-PCR)

The RNA level of ANRIL, HMGB and miR-181a in PANC-1 cells and BxPC-3 cells was measured with qRT-PCR. Total RNA was extracted using Trizol RNA isolation Kit. qRT-PCR were performed using Transcriptor First Strand cDNA Synthesis Kit (Fermentas, USA). The results were standard by the expression level of β-actin (internal reference for HMGB) or U6 (internal reference for ANRIL or miR-181a) and the average were analyzed by 2^−ΔΔCt^ method.

### Invasion assay

Cells were cultured onto basement membrane matrix on transwell cab of a 24-well culture plate (Invitrogen, Thermo Fisher Scientific, USA) without fetal bovine serum. After 48 hours, methanol (Sigma, USA) was used to fix invading cells, and the non-invaded cells were gently removed with a wet cotton swab. Crystal violet (Sigma, USA) stained, counted, and imaged.

### Cell transfection and siRNA knockdown of ANRIL

Usually, the shRNA specific for ANRIL was chemo synthesized by Genepharma (Shanghai, China). Then, the cell transfection was performed according to the instructions of Lipofectamine^™^ 2000 (Invitrogen, Thermo Fisher Scientific, USA). The detailed information of the siRNA sequence was described as follows: TTATGCTTTGCAGCACACTGG.

### Statistical analysis

All statistical data were conducted using software SPSS 16.0. Measurement data were shown as mean ± standard deviation (± s). enumeration data. *P* < 0.05 was considered statistically significant.

## RESULTS

### ANRIL and HMGB1 are highly expressed in pancreatic cancer

**Figure 1:**
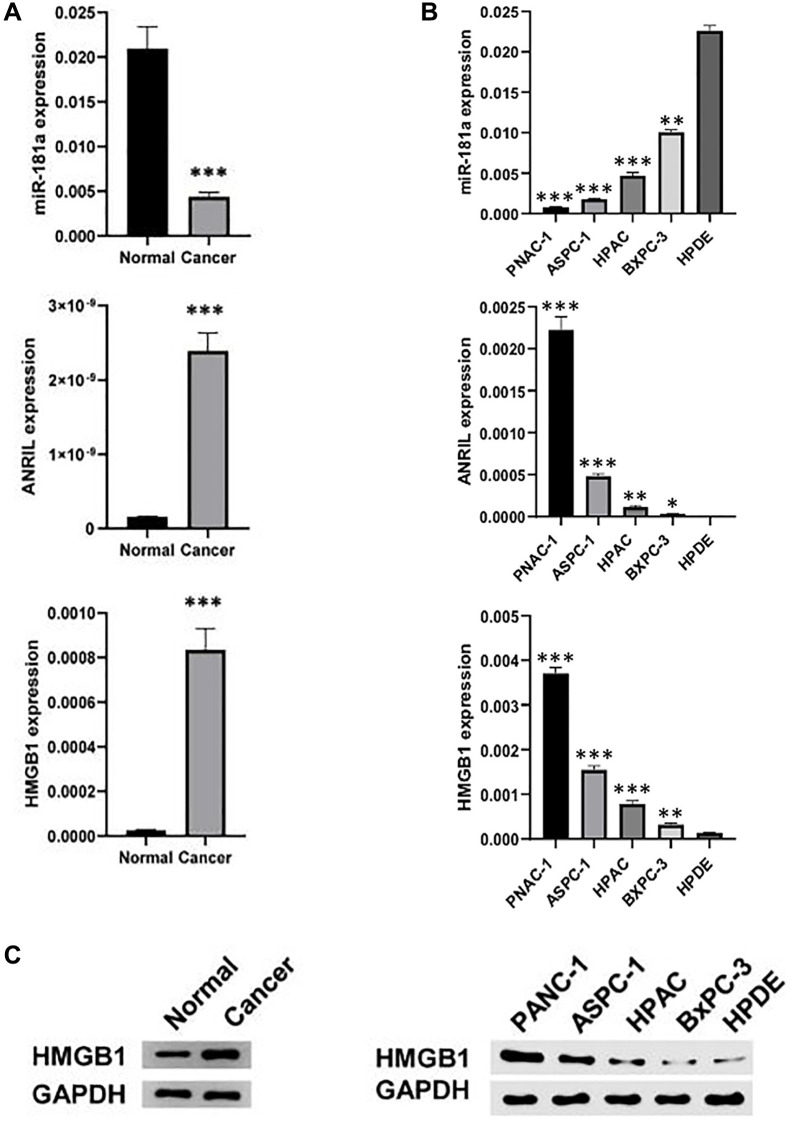
**ANRIL and HMGB1 are over-expressed in pancreatic cancer.** (**A**) qRT-PCR and western blot analyzed the expression of ANRIL, HMGB and miR-181a in human pancreatic cancer and adjacent tissues (*n* = 5). (**B**) qRT-PCR and (**C**) western blot analyzed the expression of ANRIL, HMGB and miR-181a in a series of human pancreatic cancer cell lines (PANC-1, ASPC-1, HPAC, BxPC-3) and normal pancreatic cells (HPDE). Standardized data with GAPDH. Student’s *t* test and analysis of variance compared the difference in A, B. ^*^P < 0.05, ^**^P < 0.01, ^***^P < 0.001.

The expression levels of ANRIL and HMGB1 in pancreatic cancer was firstly examined by us, and we collected 5 pairs of pancreatic and precancerous tissues. The results of qRT-PCR and western blot showed that the expression of ANRIL and HMGB1 was obviously higher in pancreatic cancer tissues than that in adjacent tissues (Figure 1A). Accordingly, they were also highly expressed in pancreatic cancer cell lines (PANC-1, ASPC-1, HPAC, BxPC-3) compared to normal pancreatic cells (HPDE) (Figure 1B). Interestingly, contrary to its above results, miR-181a was significantly lower in both pancreatic cancer tissues and cell lines (Figure 1C), which indicate that miR-181a may be regulated by ANRIL, which promotes the expression of HMGB1.

### Effects of ANRIL knockdown on proliferation, invasion, and migration of pancreatic cancer cells by promoting autophagy

**Figure 2:**
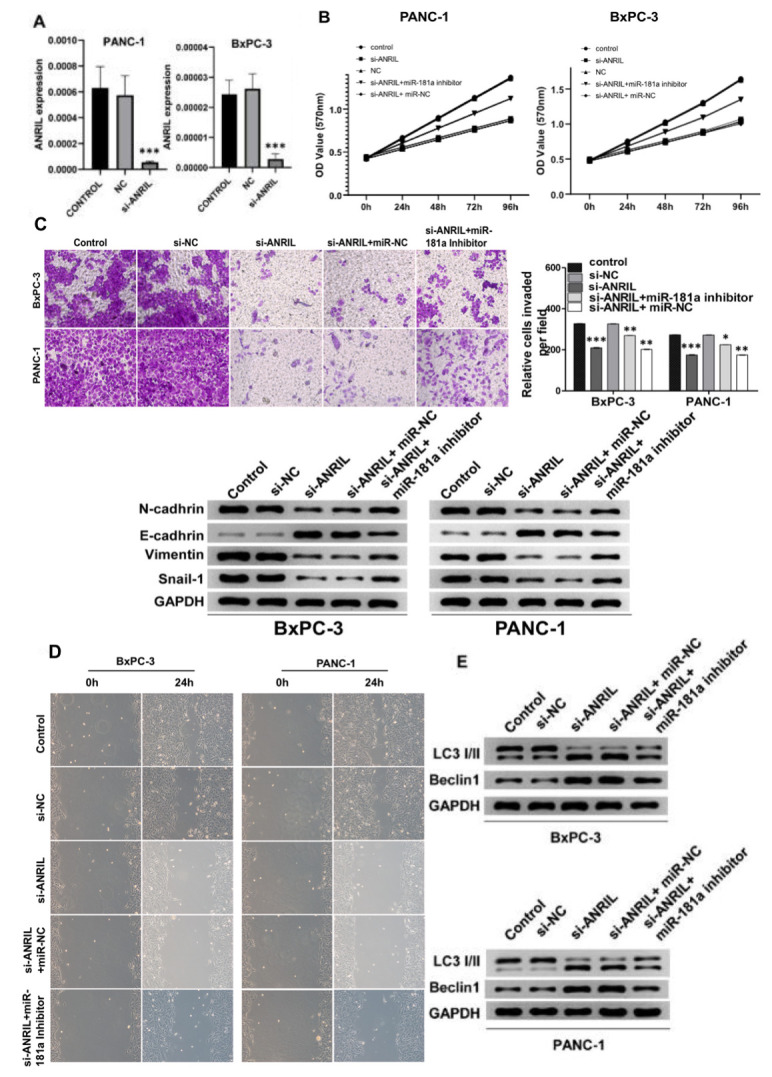
**Knockdown of ANRIL inhibits the progression of pancreatic cancer.** (**A**) Knockdown of ANRIL effectively inhibited ANRIL expression in PANC-1 cells and BxPC-3 cells. (**B**) MTT assay showed that the proliferation effect of pancreatic cancer cells transfected with si-ANRIL was significantly lower than that of the control group, while miR-181a inhibitor reversed the effect of si-ANRIL on pancreatic cancer cells. (**C**) Top: The transwell experiment reflected the effects of si-ANRIL and miR-181a inhibitor on the invasion ability of PANC-1 cells and BxPC-3 cells. Bottom: Western blot analyzed the expression levels of N-cadherin, E-cadherin, Vimentin, and Snail-1, they also represented the invasion ability of PANC-1 cells and BxPC-3 cells. (**D**) Scratch experiments revealed changes in healing ability of PANC-1 cells and BxPC-3 cells transfected with si-ANRIL, si-ANRIL+ miR-NC, si-ANRIL+miR-181a inhibitor, respectively. (**E**) The expression levels of LC3 I/II and Beclin1 reflected the regulation of autophagy levels of PANC-1 cells and BxPC-3 cells by si-ANRIL and miR-181a inhibitor. Standardized data with GAPDH. ^*^P < 0.05, ^**^P < 0.01, ^***^P < 0.001: si-NC, control siRNA; miR-NC, miRNA inhibitor control.

To investigate the role of ANRIL on the proliferation and invasion of pancreatic cancer cells, we suppressed the expression of ANRIL in PANC-1 and BxPC-3 cells by si-RNA knockdown respectively (Figure 2A). The results showed that ANRIL knockdown significantly reduced the proliferation of PANC-1 and BxPC-3 cells in MTT experiments. In contrast, when we added miR-181a inhibitor to PANC-1 and BxPC-3 cells with transfected si-ANRIL, the number of pancreatic cancer cells was higher than that of the si-ANRIL group (Figure 2B). Accordingly, transwell and migration assays showed that miR-181a inhibitor reversed the inhibitory role of si-ANRIL on the invasiveness and migration of pancreatic cancer cells (Figure 2C and 2D). Changes in the expression of cell adhesion-related proteins also illustrated the same conclusion (Figure 2C). It suggested that the inhibitory role of ANRIL on pancreatic cancer cells was mediated by miR-181a. We speculated that ANRIL- miR-181a axis restrained the activity of pancreatic cancer cells by inducing cell autophagy, so we tested the change of autophagy proteins which caused by ANRIL and miR-181a on pancreatic cancer cell. The results were consistent with our predictions that si-ANRIL and miR-181a mimics promoted the expression of LC3 II and Beclin1, while miR-181a inhibitors reversed the inhibition of autophagy by si- ANRIL (Figure 2E).

### miR-181a stimulates autophagy while suppresses cell viability of pancreatic cancer cells *in vitro*

**Figure 3:**
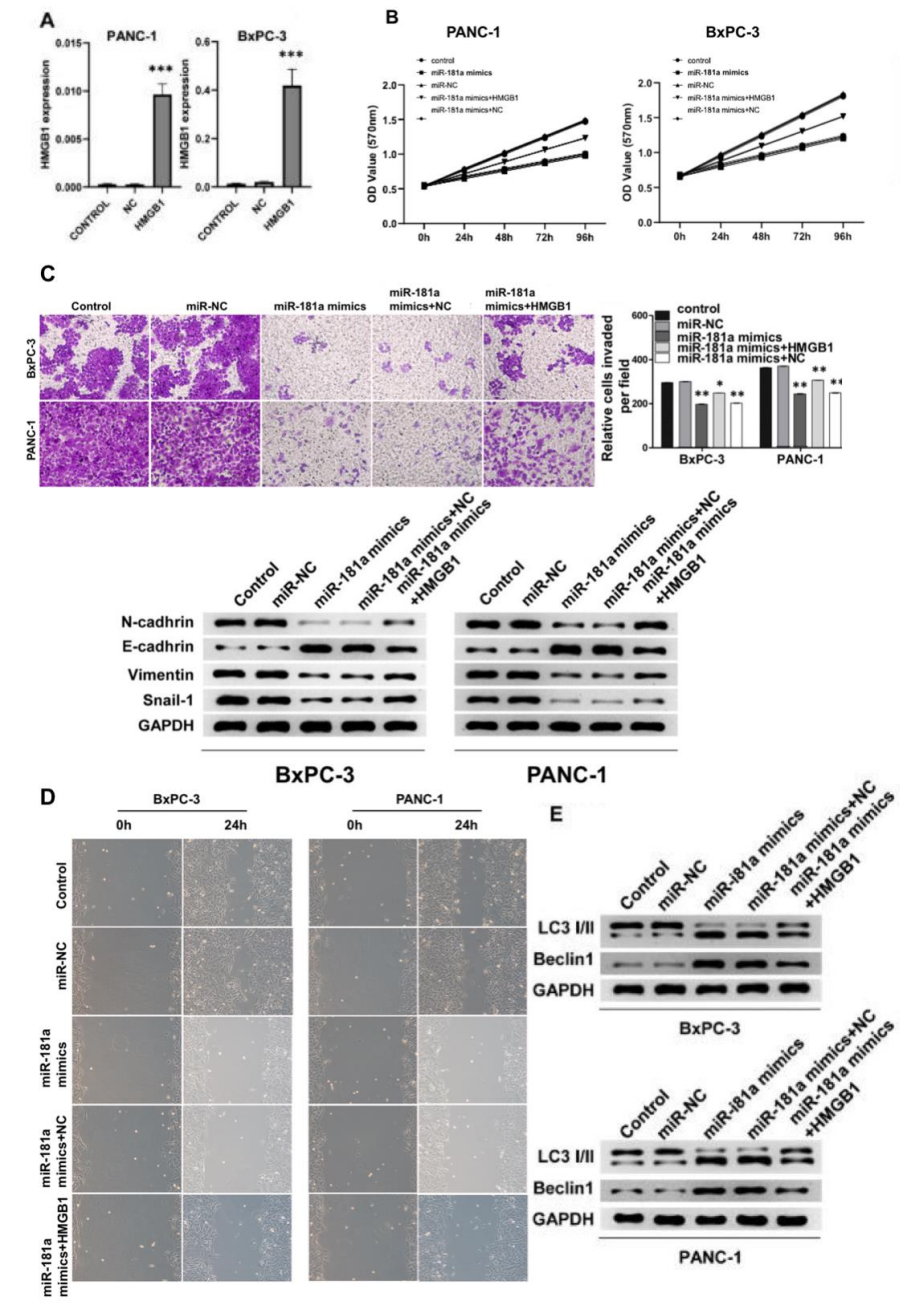
**miR-181a inhibited pancreatic cancer cell viability by promoting autophagy of pancreatic cancer cells *in vitro*.** (**A**) qRT-PCR detected the overexpression efficiency of HMGB1 in PANC-1 cells and BxPC-3 cells. (**B**) The cell proliferation of pancreatic cancer cells transfected with miR-NC, miR-181a mimics, miR-181a mimics+HMGB1, miR-181a mimics+NC was determined by MTT experiments due to their respective OD value. (**C**) Top: The transwell experiment suggested the effects of miR-181a and HMGB1 on invasion of PANC-1 cells and BxPC-3 cells, respectively. Bottom: The protein expression of N-cadherin, E-cadherin, Vimentin, and Snail-1 reflected the invasion ability of PANC-1 cells and BxPC-3 cells. (**D**) The scratch experiments suggested miR-181a inhibited the healing ability of PANC-1 cells and BxPC-3 cells, however, HMGB1 reversed this effect. (**E**) LC3 I/II and Beclin1 demonstrated the regulation of autophagy of PANC-1 cells and BxPC-3 cells by miR-181a and HMGB1. GAPDH was set as the internal control. ^*^P < 0.05, ^**^P < 0.01, ^***^P < 0.001.

We further transfected miR-181a mimics in PANC-1 and BxPC-3 cells and the transfection efficiency in both cells lines was evaluated (Figure 3A). The MTT calorimetry results suggested that miR-181a mimics significantly inhibited the cell proliferation of PANC-1 and BxPC-3 cells (Figure 3B). However, when HMGB1 was over-expressed at the same time, the activity of PANC-1 and BxPC-3 cells was partially restored, and over-expression of HMGB1 suppressed the effect of miR-181a on pancreatic cancer cells. Correspondingly, analysis of western blot showed that miR-181a inhibited the expression of adhesion proteins such as N-cadherin, E-cadherin, Vimentin and Snail-1 in pancreatic cancer cells. (Figure 3C). Then, transwell and scratch-wound assays were used to determine the effect of miR-181a on cell invasion and migration. The results showed that over-expression of miR-181a significantly inhibited cell invasion and migration of PANC-1 and BxPC-3 cells compared with that of the controls (Figure 3D). We further investigated the biological function of miR-181a and HMGB1 on autophagy of pancreatic cancer cells. Consistent with the above results, overexpression of miR-181a promoted the expression of autophagy proteins LC3 and Beclin1 in PANC-1 and BxPC-3 cells, while HMGB1 overexpression significantly reversed this trend (Figure 3E). Taken together, these results indicated that miR-181a has tumor-inhibiting activity in pancreatic cancer cells, and the biological effect of HMGB1 present the opposite effect.

### ANRIL-miR-181a-HMGB1 axis plays a critical role in the progression of pancreatic cancer

**Figure 4:**
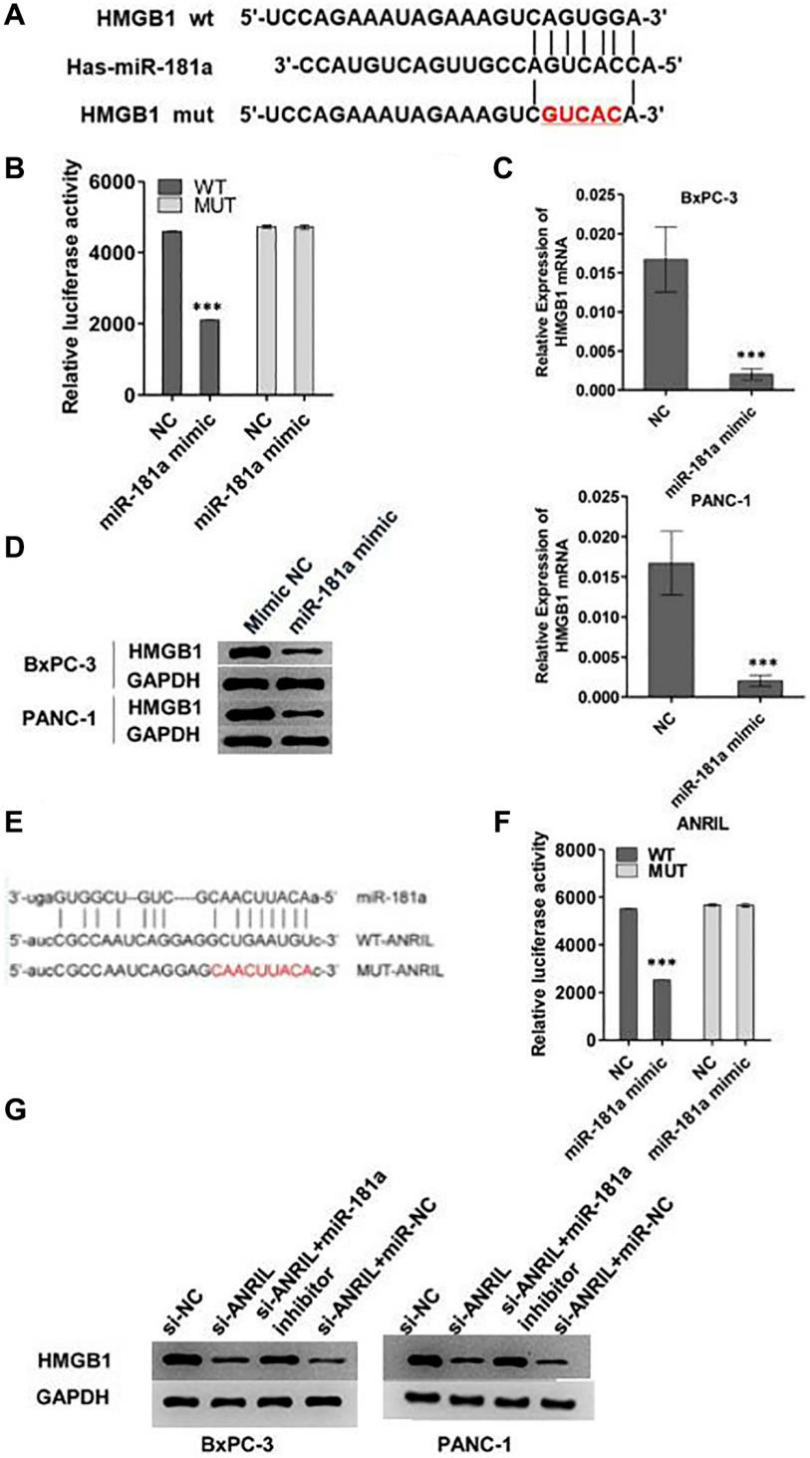
**ANRIL-miR-181a-HMGB1 axis is critical for the progression pancreatic cancer.** (**A**) The image reflected the binding sequence between miR-181a and HMGB1, and the corresponding mutant sequence between them. (**B**) Relative double luciferase activity experiment reflected the regulation of wild type HMGB1 and mutant HMGB1 activity by miR-181a. (**C**, **D**) Western blot and qRT-PCR analyzed the HMGB1 expression level which caused by miR-181a. (**E**) The image reflected the binding sequence of miR-181a and ANRIL, and mutant sequence between them. (**F**) Relative double luciferase activity experiment reflected the regulation of wild type ANRIL and mutant ANRIL activity by miR-181a. (**G**) The results of ANRIL mRNA and protein level indicated that it was regulated by miR-181a. ^*^P < 0.05, ^**^P < 0.01, ^***^P < 0.001.

Since HMGB1 could reverse the effect of miR-181a on pancreatic cancer cells, we further explored the relationship between them. We cloned the promoter region of HMGB1 into a double luciferase reporter vector, and then mutated the predicted binding region of HMGB1 to miR-181a, and the diagram of wild type (WT) and mutant (MUT) was illustrated in Figure 4A. Analysis of relative luciferase activity showed that miR-181a significantly repressed the relative luciferase activity of WT reporter, however, when MUT reporter was concerned, it did not response to miR-181a mimics compared to NC (Figure 4B). qRT-PCR and western blot analysis showed that overexpression of miR-181a down-regulated HMGB1 expression at the mRNA level and protein level in BxPC-3 and PANC-1 cells *in vitro* (Figure 4C, 4D).

To explore the role of ANRIL in this pathway, we mutated the binding site of ANRIL to miR-181a which was shown in Figure 4E. Consistent with the above results, miR-181a mimics inhibited the relative luciferase activity of WT ANRIL, while there was no response of MUT ANRIL to miR-181a mimics (Figure 4F). Western blot and qRT-PCR analysis showed that si-ANRIL decreased the expression of HMGB1 in BxPC-3 and PANC-1 cells, while miR-181a inhibitor reversed this effect (Figure 4G). These results indicated that ANRIL regulated the expression of HMGB1 by inhibiting the activity of miR-181a in pancreatic cells.

### ANRIL increases the chemotherapy resistance to gemcitabine via miR-181a/HMGB1 pathway in pancreatic cancer cells

**Figure 5:**
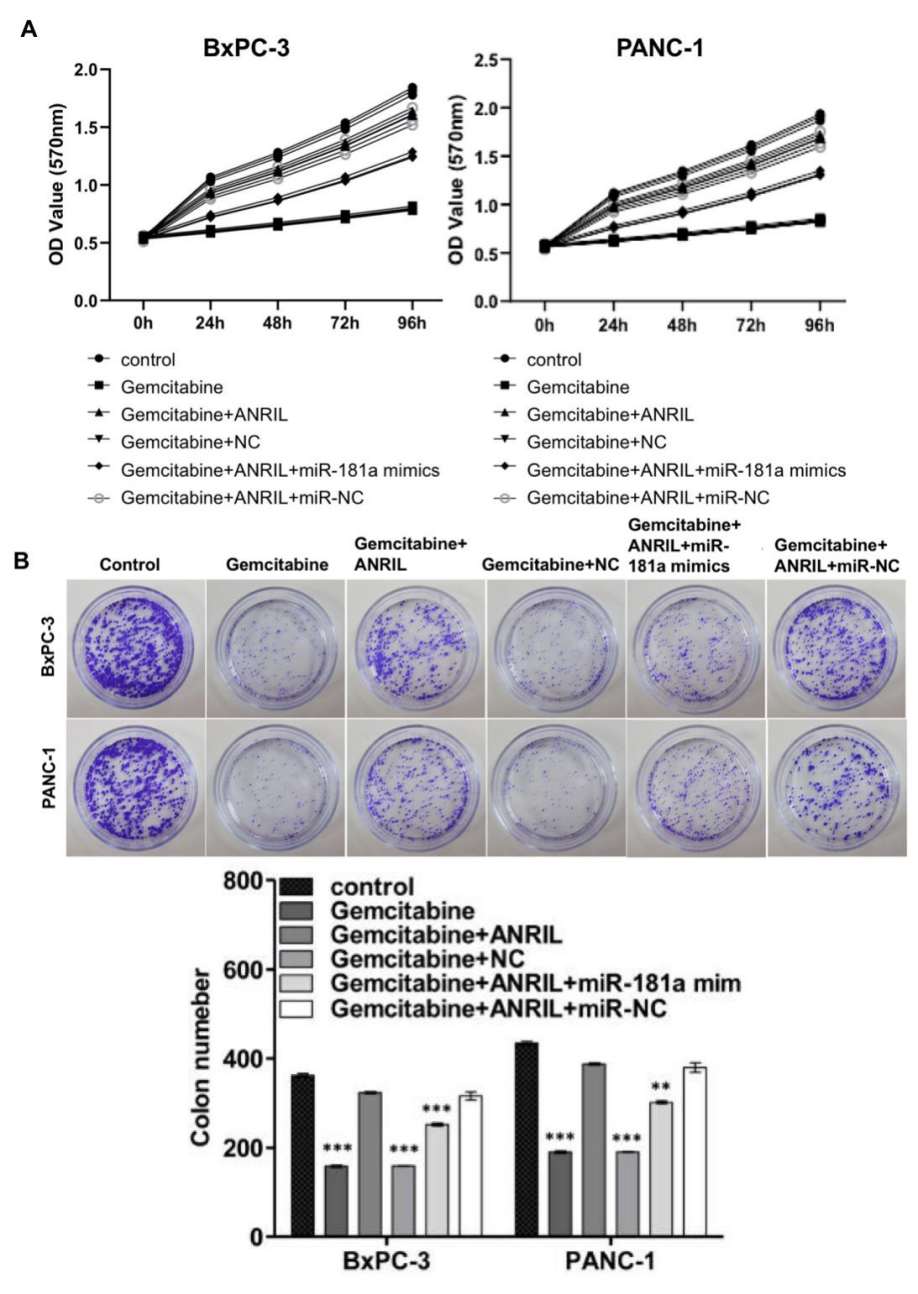
**ANRIL increases resistance of pancreatic cancer cells to gemcitabine chemotherapy.** (**A**) MTT assay showed that overexpression of ANRIL resisted the inhibition of pancreatic cancer cells by gemcitabine, but miR-181a mimics reversed the effect which caused by ANRIL. OD value reflected the relative proliferative activity of each group. (**B**) Colony formation assays suggested the effects of miR-181a, ANRIL and gemcitabine on the proliferation of PANC-1 cells and BxPC-3 cells. ^*^P < 0.05, ^**^P < 0.01, ^***^P < 0.001.

To examine the effect of ANRIL-miR-181a-HMGB1 axis on gemcitabine chemotherapy for pancreatic cancer, we tested the effects of gemcitabine and the pathway on the proliferation of pancreatic cancer cells. MTT assays and colony formation assays suggested that gemcitabine significantly restrained the proliferation of pancreatic cancer cells, and overexpression of ANRIL resisted the inhibitory effects of gemcitabine. In contrast, miR-181a mimics attenuated this antagonistic effect which caused by ANRIL in PANC-1 cells and BxPC-3 cells (Figure 5A, 5B). This means that inhibiting ANRIL maybe increase the sensitivity of pancreatic cancer cells to chemotherapy through the miR-181a / HMGB1 pathway.

## DISCUSSION

It has illustrated that lncRNAs possess pivotal roles in occurrence of cancers, such as melanoma, lung cancer, glioblastoma, and esophageal adenocarcinoma, nasopharyngeal carcinoma, gastric cancer and so forth [7–9, 14]. The mechanism of lncRNAs in recent years are growing concern, but it is still far from elucidated in tumorigenesis of pancreatic cancer. Researches showed that ANRIL could promote the development and progression of many tumors, but there was no report focusing on the activity of ANRIL in the chemotherapy resistance of pancreatic cancer. We firstly reported that ANRIL could promote the tumor cell proliferation, invasion, migration, and exacerbates chemotherapy resistance to gemcitabine of pancreatic cancer by managing miR-181a/ HMGB1 axis and restraining cell autophagy, which reveal the role of ANRIL and miR-181a in therapy of pancreatic.

ANRIL was famous for its huge non-protein coding region in which the 21 exons could be transcribed into different kinds of circular or linear isoforms [15]. It has reported that ANRIL was not only obviously up-regulated, but promoted proliferation and invasive ability through regulation of miR-181a in cervical cancer [16]. We found that ANRIL was up-regulated in the tissues of pancreatic cancer, while found that ANRIL promotes cell proliferation, invasion, migration in PANC-1 and BxPC-3 cells, as well as enhances chemotherapy resistance to gemcitabine, while knockdown of ANRIL decreased those effects.

miR-181a will take part in virous function of cells including growth, proliferation, survival, death and maintenance [17, 18]. Accumulating evidence confirms that the interactions between lncRNAs and miRNAs will affect post-transcriptional regulation [19]. Research before had indicated that miR-181a could restrain migration and carcinogenesis of breast and colon cancer cells by down-regulating MMP-14 [20]. The interaction between ANRIL and miR-181a had been clarified in laryngeal squamous cell carcinoma, which was negatively correlated [17]. In our research, we further confirmed this conclusion in pancreatic cancer. Overexpression of ANRIL could obviously promote the cell viability and proliferation of PANC-1 and BxPC-3 cells, while after ANRIL knockdown, the cell proliferation was inhibited, so was the invasion and migration. While overexpression of miR-181a reversed that effect.

But, the further mechanisms of miR-181a on cellular behaviors still remained largely unknown. Accumulating evidence showed that interaction of miRNA and HMGB1 involved in EMT, cell apoptosis, and autophagy [21–23]. And previous studies proved that HMGB1 can induce autophagy [24, 25]. We demonstrated that overexpression of miR-181a decreased LC3 I/II but increased Beclin1, which indicated that autophagy was activated. When HMGB1 overexpressed, the result was opposite [26]. To make the relationship between miRNA and HMGB1 clear, we mutated 3′UTR of HMGB1, then we concluded that miR-181a directly targeted to 3′UTR of HMGB1 to activate autophagy. Maybe it is still possible that miR-181a indirectly affects HMGB1 expression, although the 3′UTR has a potential binding site.

In summary, we presented the potential effect of ANRIL and miR-181a on pancreatic cancer in this research. The results indicated that ANRIL was up-regulated but miR-181a was declined in pancreatic cancer. Inhibition of ANRIL could restrain the proliferation, invasion, migration of cells, and reduce chemotherapy resistance to gemcitabine by targeting miR-181a to stimulate HMGB1-induced cell autophagy. This study will provide new insights for ANRIL and miR-181a and potential targets for the therapy of pancreatic cancer.
